# Next-Generation Lung Cancer Surgery: A Brief Trip into the Future of the Research

**DOI:** 10.3390/jcm12103479

**Published:** 2023-05-15

**Authors:** Clarissa Uslenghi, Monica Casiraghi, Lorenzo Spaggiari, Luca Bertolaccini

**Affiliations:** 1 Department of Thoracic Surgery, IEO, European Institute of Oncology IRCCS, 20141 Milan, Italy; clarissa.uslenghi@unimi.it (C.U.); monica.casiraghi@ieo.it (M.C.); lorenzo.spaggiari@ieo.it (L.S.); 2 Department of Oncology and Hemato-Oncology, University of Milan, 20122 Milan, Italy

Lung cancer is the third most frequent cancer and the leading cause of cancer-related mortality worldwide [1]. Non-Small-Cell Lung Cancer (NSCLC) represents 80% of all lung cancers. The lack of an effective screening system and estimated 5-year overall survival rate of less than 25%, if one considers all tumor stages, mean that it is a frequent subject of many scientific articles. According to PubMed, 21,666 articles have been published in the last five years with NSCLC as a keyword, demonstrating that scientific research is still active and productive.

“*Space/the final frontier/these are the voyages of the Starship Enterprise/its five-year mission/to explore […]/to boldly go where no man has gone before*”. With these words, we are taken to the future of Star Trek, a series that would profoundly mark the science fiction imagination. Since this phrase was introduced with the silhouette of the U.S.S. Enterprise, that is, the introduction of Star Trek, it has become one of the most famous expressions of science fiction. Similar to the trip of the U.S.S. Enterprise, over the past two years, highly cited articles have been published in the *Journal of Clinical Medicine* with the aim of describing the possible future scenarios of lung cancer surgery. These articles were widely read and became highly influential within the field. Therefore, we take a brief trip among these relevant articles and briefly comment on them.

The standard radical treatment for early-stage NSCLC consists of anatomic lobectomy and hilum-mediastinal lymph node dissection [2]. The literature widely demonstrates that the surgical approach has better oncological outcomes than stereotactic body radiation therapy in the population with early-stage NSCLC, even for elderly patients [3]. The goal of a surgical excision should be a microscopically complete resection (R0), defined by the published guidelines as tumor resection, free resection margins, and lymph node (LN) dissection [4]. This has led to the need to make surgery as accessible and non-invasive as possible. For these reasons, minimally invasive surgery has become the standard surgical approach, and many studies have been conducted on the possibility of reducing resected lung volumes while maintaining oncological radicality.

The role of sub-lobar resection, including both anatomical segmentectomy and wide-wedge resections, remains controversial. Several studies have recently been published to evaluate the feasibility and safety of sub-lobar resections. Kraeve et al. analyzed 10-year outcome data of the Surveillance, Epidemiology, and End-Results (SEER) database, showing that patients with a tumor size < 3 cm (cT1N0, stage IA) who underwent lobectomy had significantly better survival rates than segmentectomy patients [5]. A systematic review by De Zoysa et al. concluded that lobectomy should be performed for early-stage NSCLC in younger patients with an acceptable cardiopulmonary reserve [6]. However, the retrospective nature of these studies and the lack of preoperative risk factor adjustment may represent selection biases [7]. Two randomized trials were initiated to obtain more evidence: JCOG0802/WJOG4607L in Japan and CALBG 140503 in North America [8,9]. The Chinese randomized control trial NCT02011997 is an ongoing Chinese randomized control trial aiming to compare complete Video-Assisted Thoracoscopic Surgery (cVATS) lobectomy with cVATS segmentectomy. Sub-lobar resections were performed for small peripheral tumors, cases with impaired cardiopulmonary function [10], or elderly patients (elderly patients were defined as ≥65 years). Nonetheless, complex segmentectomies often correlate to longer operating time and prolonged air leaks and should be introduced cautiously for elderly patients [11]. According to Wang et al., segmentectomy showed comparable survival outcomes and recurrence patterns to lobectomy for the elderly, while wedge resection showed inferior outcomes. The absence of systematic lymph node dissection and the non-anatomic resection may have contributed to the elevated local recurrence rates. As a result, for now, anatomic segmentectomy with radical lymphadenectomy represents an alternative for elderly patients with early-stage NSCLC [12].

The minimally invasive surgical technique known as Robotic-Assisted Thoracoscopic Surgery (RATS), which represents a technological evolution of the VATS procedure, has rapidly expanded into the surgical practice of thoracic surgeons. Over the years, the application of robotic thoracic surgery has also been extended to high-risk patients. Zirafa et al. evaluated high-risk patients with the ASA-PS score and then stratified them according to perioperative risk. The results showed that for high-risk NSCLC patients, lung resection via the robotic approach could represent a safe therapeutic option in terms of the short-term postoperative outcomes and oncological results [13]. Compared to VATS, robotic technique seems to have some technical advantages, such as a better view of the operative field, more straightforward use of the instruments, and the execution of more precise and complex movements [14]. The latest literature results show that the robotic approach results in less blood loss, lower conversion and complication rates, and an improved lymph node harvest compared to thoracoscopy [15]. An adequate lymphadenectomy has a crucial role in the oncological outcomes of NSCLC patients, preventing downstaging and undertreatment. Gallina et al. showed that the most relevant indicators of lymphadenectomy quality are the number of dissected hilar and mediastinal nodal stations and the number of harvested nodes, confirming that at least ten lymph nodes must be dissected to obtain a proper staging, as reported in the current recommendations [16].

Together with the tumor size and local extension, lymph node metastases are crucial prognostic factors influencing therapeutic regimens and survival [17]. In particular, proper mediastinal staging is fundamental in preoperative evaluation. F-fluorodeoxyglucose positron emission tomography/computed tomography (18F-FDG PET/CT) is routinely used as a preoperative non-invasive staging method of lymph node status for lung cancer [18]. The literature shows that the downstaging of LNM based on PET/CT findings was observed in almost 14% of patients, and upstaging was observed in 8%. The preoperative PET/CT and histopathological findings matched almost 79% of the patients [19].

N2 stage III NSCLC patients have a poor prognosis with a high incidence of distant metastasis or local tumor recurrence [20]. The extra-nodal extension (ENE) of the involved lymph nodes indicates the presence of cancer cells extending throughout the lymph node capsule into the surrounding fibrous adipose tissue. ENE is already a well-recognized prognostic factor for different solid tumors, but only a few recent studies have reported data regarding the importance of ENE in NSCLC. Patients with ENE had more advanced pathological stages and frequently underwent pneumonectomy. Recent studies reported that ENE also seems to have a strong association with recurrence and mortality in NSCLC, representing the most meaningful predictor of survival regardless of the histologic cell type [21].

Additionally, with respect to N2 patients, recent studies based on the SEER database showed that postoperative radiotherapy (PORT) alone or combined with postoperative chemotherapy could prolong survival [22]. However, even if an optimal form of adjuvant radiation therapy for these patients has not yet been established, radiotherapy is considered as a valid option for patients with an increasing extent of mediastinal nodal disease. In contrast, PORT may not improve survival rates among elderly patients over 75 years [23].

In conclusion, NSCLC remains a significant global health issue, with a poor 5-year overall survival rate. Even in the near future, anatomic lobectomy and hilum-mediastinal lymph node dissection will represent the standard treatment for early-stage NSCLC. Minimally invasive surgery, such as RATS, has evolved and demonstrated benefits over traditional thoracoscopy. However, sub-lobar resection, such as segmentectomy, remains controversial and is usually performed in cases with impaired cardiopulmonary function or elderly patients [24]. An adequate lymphadenectomy is essential for the oncological outcomes of NSCLC patients. In the preoperative non-invasive staging of lymph node status for lung cancer, 18F-FDG PET/CT is routinely used. Although N2 stage III NSCLC patients have a poor prognosis, ENE in the involved lymph nodes indicates the presence of cancer cells extending throughout the lymph node capsule into the surrounding soft tissue. The increasing number of articles published on NSCLC shows that scientific research on this subject is still active and productive, and there is a pressing need for further research on NSCLC treatment.
